# A Chloroplast-Localized Rubredoxin Family Protein Gene from *Puccinellia tenuiflora* (*PutRUB*) Increases NaCl and NaHCO_3_ Tolerance by Decreasing H_2_O_2_ Accumulation

**DOI:** 10.3390/ijms17060804

**Published:** 2016-05-30

**Authors:** Ying Li, Panpan Liu, Tetsuo Takano, Shenkui Liu

**Affiliations:** 1 Key Laboratory of Saline-alkali Vegetation Ecology Restoration in Oil Field (SAVER), Ministry of Education, Alkali Soil Natural Environmental Science Center (ASNESC), Northeast Forestry University, Harbin Hexing Road, Harbin 150040, China; ly7966@163.com (Y.L.); belle.pp.liu@gmail.com (P.L.); 2 Asian Natural Environmental Science Center, University of Tokyo, Nishitokyo-shi, Tokyo 188-0002, Japan; takano@anesc.u-tokyo.ac.jp

**Keywords:** rubredoxin family protein, *Puccinellia tenuiflora*, ROS

## Abstract

Rubredoxin is one of the simplest iron–sulfur (Fe–S) proteins. It is found primarily in strict anaerobic bacteria and acts as a mediator of electron transfer participation in different biochemical reactions. The *PutRUB* gene encoding a chloroplast-localized rubredoxin family protein was screened from a yeast full-length cDNA library of *Puccinellia tenuiflora* under NaCl and NaHCO_3_ stress. We found that *PutRUB* expression was induced by abiotic stresses such as NaCl, NaHCO_3_, CuCl_2_ and H_2_O_2_. These findings suggested that *PutRUB* might be involved in plant responses to adversity. In order to study the function of this gene, we analyzed the phenotypic and physiological characteristics of *PutRUB* transgenic plants treated with NaCl and NaHCO_3_. The results showed that *PutRUB* overexpression inhibited H_2_O_2_ accumulation, and enhanced transgenic plant adaptability to NaCl and NaHCO_3_ stresses. This indicated *PutRUB* might be involved in maintaining normal electron transfer to reduce reactive oxygen species (ROS) accumulation.

## 1. Introduction

Nowadays, soil alkalinization is very severe. Salinity and alkalinity are major environmental stresses on soil that limit crop distributions and yields worldwide [1,2,3]. In Northeast China, the total area of alkaline saline soil is about 3.73 × 10^6^ ha and is expanding at a rate of 1.4% annually, making it one of the three largest sodic-saline areas in the world [4,5]. In such soil, plants suffer adverse stress factors, such as Na^+^ and HCO_3_^−^/CO_3_^2−^ ions, and high pH. These factors can directly affect nutrient uptake, organic acid balance, and ion homeostasis at a whole-plant level [6,7,8,9]. Research shows that only a small number of plants can complete their entire life cycle in highly alkaline areas. *Puccinellia tenuiflora*, a graminaceous and alkali-tolerant halophyte species, is one such plant. It can complete its life cycle in highly alkaline soil (pH 10). *P. tenuiflora* has evolved various strategies to adapt to saline or alkaline stress, such as maintaining ion balance, osmotic homeostasis adjustment, and removal of reactive oxygen species (ROS) [10,11,12,13,14,15]. Thus, it is considered a saline-alkali soil pioneer plant. To date, several stress-responsive genes in *P. tenuiflora* have been isolated and their biological functions have been tested [16,17,18,19,20,21]. *P. tenuiflora* is also considered an ideal model plant for discovering resistance genes and studying the resistance mechanism of halophytes.

Rubredoxin is a kind of non-heme iron protein that contains a [Fe(SCys)_4_] center. It is one of the simplest iron–sulfur (Fe–S) proteins, containing a single Fe atom and four S atoms [22]. Rubredoxin is mostly found in strict anaerobic bacteria, including bacteria, archaebacteria and a few microaerobic bacteria in nature [23,24,25,26,27]. Relatively few of these proteins have been found in plants. Research suggests rubredoxin proteins act as mediators of electron transfer for various enzymes in anaerobic bacteria [24,28,29,30]. Recently, a new pathway called the Super Oxide Reductase reaction(SOR) was identified that provides an alternative superoxide reduction pathway in anaerobic microorganisms. Rubredoxins of various anaerobes appear to act as an electron donor in the SOR reaction to reduce the production of O_2_ [31,32,33].

In our previous work, we identified some candidate salt-responsive genes in *P. tenuiflora* using the Full-length cDNA Over-eXpressor gene (FOX)-hunting system. This system is a very effective tool for plant functional gene research that does not require any knowledge of the genome of interest or genetic mapping. It has opened up novel approaches for elucidating the functions of genes that control metabolic pathways and determine plant morphological characteristics [34,35]. *PutRUB* was one of the genes we identified. In this study, we analyzed its expression under different stress conditions. The function of *PutRUB* in NaCl and NaHCO_3_-stress conditions was investigated in Arabidopsis transgenic the *PutRUB* gene. We hope these results will provide a theoretical basis for future exploration of the adaptation mechanism of *P. tenuiflora*.

## 2. Results and Discussion

### 2.1. Characteristics of PutRUB

The full-length cDNA of *PutRUB* was 1005 bp, with a 5′-UTR of 52 bp, an open reading frame (ORF) of 774 bp, and a 3′-UTR of 179 bp. From the ORF, we deduced that *PutRUB* encoded a 225-amino acid protein with a predicted molecular mass of 27.38 kDa and a theoretical *pI* of 9.85. The sequence contained an N-terminal signaling peptide with the most likely cleavage site between positions 22 and 23 (SHC-AD).

**Figure 1:**
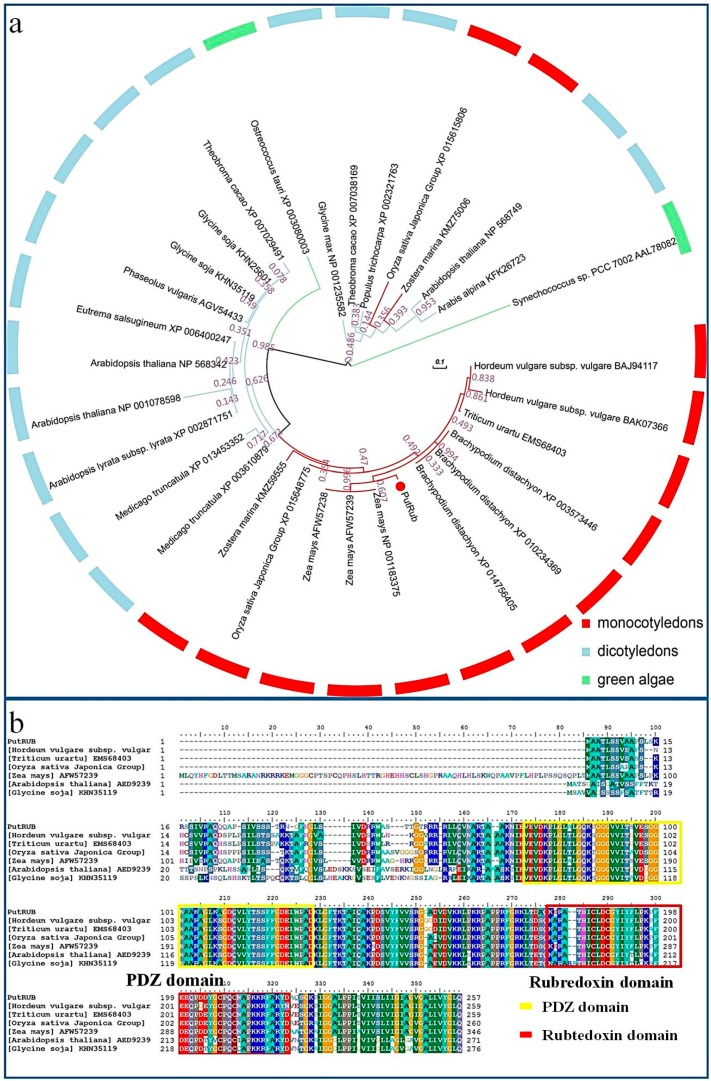
Phylogenetic analysis of *PutRUB*. (**a**) Amino acid sequence of *PutRUB* and phylogenetic trees analysis of 30 rubredoxin family protein sequences in plants; (**b**) The analysis of the amino acid sequence of *PutRUB* with rubredoxin family protein in *Triticum urartu*, *Horeum vulgare*, *Oryza sativa* Japonica Group, *zea mays*, *Arabidopsis thaliana* and *Glycine soja*.

To study the phylogenetic relationships of *PutRUB* and other candidate rubredoxin proteins, we selected 31 complete protein sequences from 19 species to construct a phylogenetic tree. These included 13 candidate rubredoxin protein sequences from monocotyledons (3 from *Zea mays*: NP_001183375, AFW57239, AFW57238; 1 from *Triticum urartu*: EMS68403; 2 from *Hordeum vulgare* subsp.*vulgare*: BAJ94117, BAK07366; 3 from *Brachypodium distachyon*: XP_003573446, XP_010234369, XP_014756405; 2 from *Oryza sativa* Japonica Group: XP_015648775, XP_015615806; 2 from *Zostera marina*: KMZ75006, KMZ59555), 15 candidate proteins from other plants (2 from *Medicago truncatula*: XP_003610879, XP_013453352; 3 from *Arabidopsis thaliana*: NP_568342, NP_001078598, NP_568749; 2 from *Glycine soja*: KHN35119, KHN25601; 1 from *Glycine max*: NP_001235582; 1 from *Eutrema salsugineum*: XP_006400247; 1 from *Arabidopsis lyrata* subsp. *lyrata*: XP_002871751; 2 from *Theobroma cacao*: XP_007029491, XP_007038169; 1 from *Phaseolus vulgaris*: AGV54433; 1 from *Populus trichocarpa*: XP_002321763; 1 from *Arabis alpina*: KFK26723) and two from green algae (*Synechococcus* sp. PCC 7002: AAL78082, *Ostreococcus tauri*: XP_003080003) (Figure 1a). The results showed that *PutRUB* had the closest phylogenetic relationship with *Z. mays* NP_001183375, and had close relationships with most monocotyledonous plants.

Alignment of the amino acid sequence of PutRUB with sequences from *T. urartu* (EMS68403), *H. vulgare* subsp. *vulgare* (BAJ94117), *O. sativa* Japonica Group XP_015648775), *Z. mays* (NP_001183375), *A. thaliana* (AED92391.1) and *G. soja* (KHN35119) (Figure 1b) suggested PutRUB possessed characteristics common to rubredoxin family proteins in these plants and contained two domains. In the N-terminal region, there is a putative PDZ domain; in the the C-terminus, there is a rubredoxin domain. The PDZ domain is potentially involved in interactions of protein to protein [36,37]. The conserved regions of PutRUB and the phylogenetic tree suggested that *PutRUB* belonged to the rubredoxin family, but its function have yet to be elucidated.

### 2.2. Subcellular Localization of the PutRUB: GFP Fusion Protein

**Figure 2:**
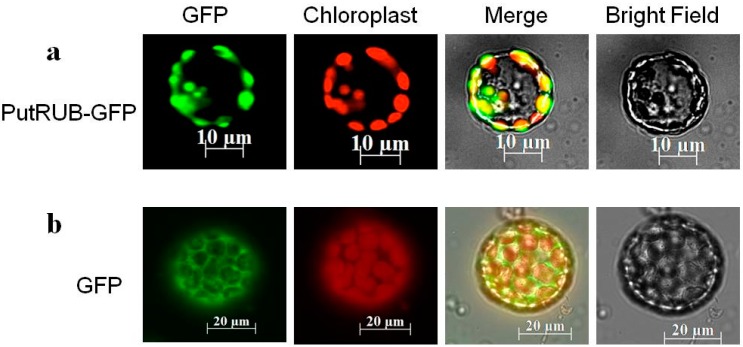
Subcellular localization of *PutRUB* in *Arabidopsis* protoplasts. (**a**) *PutRUB*: GFP within *Arabidopsis* protoplasts; (**b**) GFP within *Arabidopsis* protoplasts. The protoplasts were stained with MitoTracker Red. Scale bar = 10 µm.

To determine the accurate subcellular localization of the *PutRUB* protein in plant cells, we used transgenic plants containing *pBI121-PutRUB-GFP*. The GFP signal was stably accumulated in the chloroplast (Figure 2).

### 2.3. Expression of the PutRUB Gene Is Induced by Abiotic Stresses

**Figure 3:**
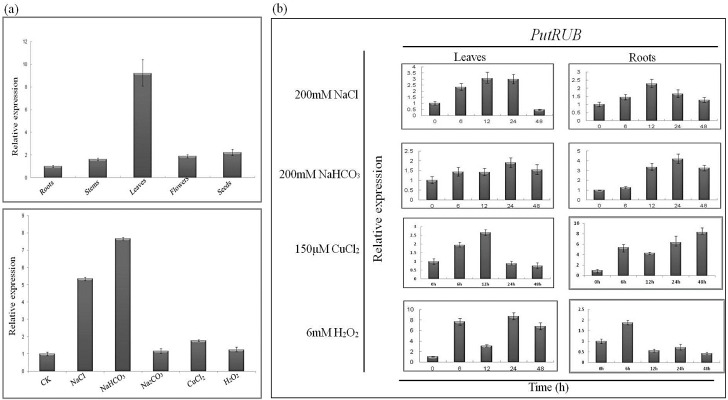
Expression pattern of the *PutRUB* gene by qRT-PCR. (**a**) *PutRUB* expression in *Puccinellia tenuiflora* tissues and *PutRUB* expression in *Puccinellia tenuiflora* seedlings under virous abiotic stress; (**b**) *Puccinellia tenuiflora* leaves and roots deal with NaCl and NaHCO_3_ stress.

The steady-state mRNA levels of *PutRUB* in different tissues and under different stresses (NaCl, NaHCO_3_, CuCl_2_ and H_2_O_2_ treatments) were assayed with Semi-quantitative Polymerase Chain Reaction (RT-PCR) (Figure 3). *PutRUB* was accumulated in all tissues that we studied (Figure 3a). The highest expression levels were in leaves, while the lowest levels were in roots, indicating that *PutRUB* expression has tissue specificity. *PutRUB* was localized in chloroplasts, similarly to a rubredoxin in *Synechococcus* sp. PCC 7002, and had the highest expression in leaves. The *Synechococcus* sp. PCC 7002 rubredoxin is localized in chloroplasts and was confirmed to be involved in the building of the interpolypeptide (4Fe–4S) cluster Fx of PSI [38,39]. The above results suggested that *PutRUB* expression may be associated with photosynthesis.

Previous studies used the *PutRUB* gene to enhance the resistance of yeast to biotic and abiotic stresses [34]. In this paper, we analyzed the pattern of expression of the *PutRUB* gene under different abiotic stresses (Figure 3a). All of the stresses induced *PutRUB* expression, but the highest expression was observed under NaCl and NaHCO_3_ stresses. Subsequently, the expression of *PutRUB* was monitored over a time course under NaCl, NaHCO_3_, CuCl_2_ and H_2_O_2_ stresses (Figure 3b). Figure 3b shows that *PutRUB* was up-regulated in both roots and leaves by exposure to 200 mM NaCl, 200 mM NaHCO_3_, 150 µM CuCl_2_ and 6 mM H_2_O_2_, indicating that *PutRUB* may be involved in responses to these stresses.

### 2.4. Response to NaCl and NaHCO_3_ Stress in PutRUB Transgenic Plants

**Figure 4:**
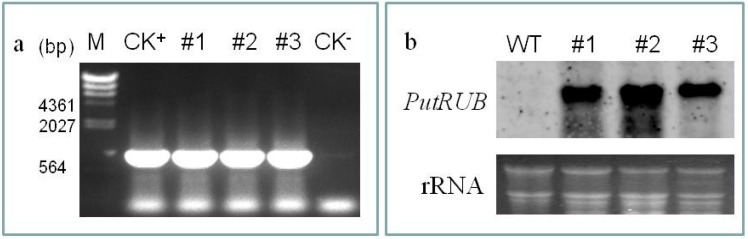
Molecular detection of *PutRUB* in transgenic *A. thaliana*. (**a**) PCR amplification T1 transgenic *A. thaliana* lines (CK^+^ is plasmids of *PutRUB*, T1-#1, #2, #3, CK^−^ is wild *A. thaliana* lines); (**b**) Northern blot was used for examination of T3 transgenic *A. thaliana* lines.

To analyze the function of *PutRUB* under NaCl and NaHCO_3_ stresses, we constructed transgenic Arabidopsis plants overexpressing *PutRUB* under the control of the strong constitutive CaMV 35S promoter. Three independent T_1_ and T_3_ generation transgenic *A. thaliana* lines were identified by PCR amplification and northern blot analysis (Figure 4). The results showed that each of these lines expressed *PutRUB*. Thus, independent transgenic plants overexpressing *PutRUB* (#1, #2, #3) were used for assays of the root length and fresh weight.

**Figure 5:**
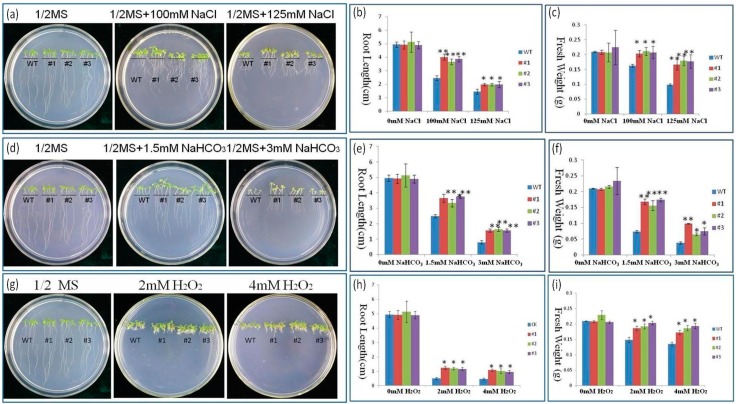
Phenotypic analyses of *PutRUB* transgenic plants treated with NaCl, NaHCO_3_and H_2_O_2_. (**a**) Phenotypes of WT and *PutRUB* transgenic seedlings treated with 150 and 125 mM NaCl; (**b**) Effects of NaCl on the root length of wild-type (WT) and *PutRUB* transgenic plants; (**c**) Effects of NaCl on fresh weight of wild-type (WT) and *PutRUB* transgenic plants; (**d**) Phenotypes of WT and *PutRUB* transgenic seedlings treated with 1.5 and 13 mM NaHCO_3_; (**e**) Effects of NaHCO_3_ on the root length of wild-type (WT) and *PutRUB* transgenic plants; (**f**) Effects of NaHCO_3_on fresh weight of wild-type (WT) and *PutRUB* transgenic plants; (**g**) Phenotypes of WT and *PutRUB* transgenic seedlings treated with 2 and 14 mM H_2_O_2_; (**h**) Effects of H_2_O_2_ on the root length of wild-type (WT) and *PutRUB* transgenic plants; (**i**) Effects of H_2_O_2_ on fresh weight of wild-type (WT) and *PutRUB* transgenic plants. Single and double asterisks represent significant differences from WT at *p* < 0.05 and *p* < 0.01, respectively.

We analyze the phenotypes (fresh weights and root lengths) of WT and transgenic seedlings in the presence and absence of NaCl and NaHCO_3_ (Figure 5a–f). Under 1/2MS medium, no differences were observed between the WT and *PutRUB* transgenic seedlings. When the NaCl and NaHCO_3_ concentration was increased, the growth of all seedlings was gradually retarded. Under stress treatments (100 and 125 mM NaCl; 1.5 and 3 mM NaHCO_3_), the root lengths and fresh seedling weights of the transgenic seedlings were significantly higher than those of the WT.

Salt and alkali stress are major abiotic stresses. To adapt to these stresses, plants have developed some sophisticated mechanisms to sense external pressure signals. Plants can change their physiological and morphological characteristics to adapt to adversity [9,40]. Multiple stresses often induce the same cell signal transduction pathways. All of these stresses will lead to a common adverse effect that is oxidative stress [41,42,43,44]. When transgenic lines were exposed to H_2_O_2_, they showed much better root and leaf growth than the WT, and their fresh weights and root lengths were higher than those of the WT (Figure 5g–i). Thus, we suspect *PutRUB* may play a vital role in reducing the damage from H_2_O_2_.

### 2.5. PutRUB Is Involved in Reducing of Reactive Oxygen Species (ROS) Detoxification

**Figure 6:**
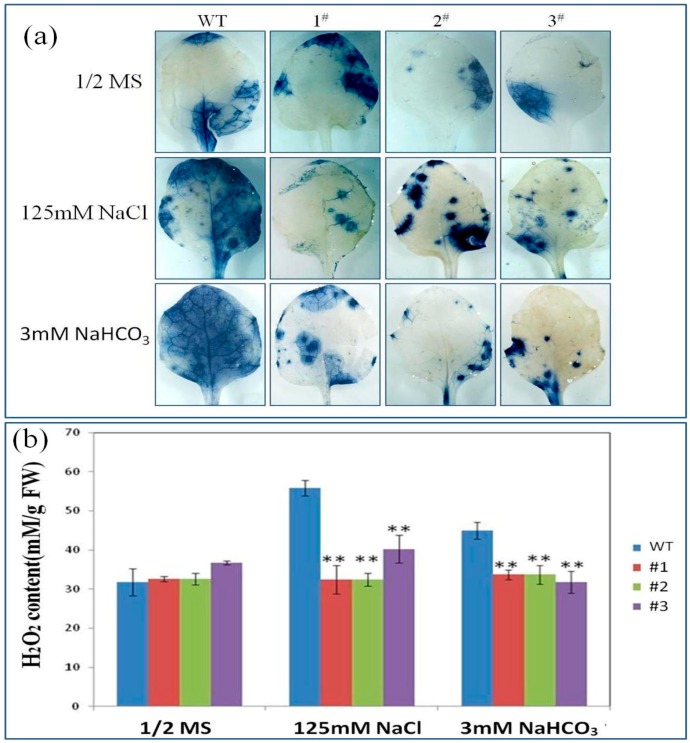
Physiological analyses of *PutRUB* transgenic seedlings treated with NaCl and NaHCO_3_. (**a**) Visualization of O_2_^−^ by NBT staining in leaves of 3-week old transgenic seedlings treated in 1/2MS, 125 mM NaCl and 3 mM NaHCO_3_ for 12 h. The blue color represents the accumulation of O_2_^−^; (**b**) H_2_O_2_ contents of 3-week-old transgenic seedlings treated with 1/2MS, 125 mM NaCl and 3 mM NaHCO_3_for 24 h. Experiments were repeated three times. Single and double asterisks represent significant differences from WT at *p* < 0.05 and *p* < 0.01, respectively.

Staining with nitroblue tetrazolium (NBT) specifically tests superoxide radicals. NBT staining demonstrated that dealing with NaCl and NaHCO_3_ induced O_2_^−^ accumulation in the leaves of *PutRUB* transgenic lines and WT plants (Figure 6a). In the presence of 100 and 125 mM NaCl, and 1.5 and 3 mM NaHCO_3_, less O_2_^−^ was accumulated in the leaves of *PutRUB* plants than in the WT. We obtained similar results for measurements of H_2_O_2_ content. As shown in Figure 6b, under NaCl and NaHCO_3_ stresses, H_2_O_2_ accumulation in *PutRUB* transgenic lines was lower than in WT plants. These results suggest that *PutRUB* is involved in decreasing H_2_O_2_ accumulation in plants and plays important roles in response to NaCl stress and NaHCO_3_ stress.

Previous reports have shown that when plants are exposed to NaCl stress or oxidative stress, H_2_O_2_ and superoxide anions increase, injuring the plant cells [45]. Rubredoxins from *Pyrococcus furiosus* and *Desulfovibrio vulgaris* play an important role in sharp contrast to the role of superoxide dismutase, effectively reducing damage from O_2_ in superoxide detoxification [31,32,33]. *AtRUB* (At5g17170) plays an important role in the control of ROS detoxification in response to NaCl stress [37]. These results all show that rubredoxins are key players in plant tolerance and responses to oxidative stress. Rubredoxins are electron carriers for various enzymes that reduce the damage from oxidative stress and protect the normal microbial life cycle [30,31,46]. Therefore, we assume that *PutRUB* maintains normal electron transfer to enhance transgenic plant tolerance and reduce ROS accumulation under NaCl and NaHCO_3_ stresses.

## 3. Materials and Methods

### 3.1. Plant Materials and Stress Treatments

Wild-type of *P. tenuiflora* plants, organs/tissues (roots, stems, leaves, flowers and seeds) and mature seeds were collected from the AnDA experimental base of the Alkali Soil Natural Environmental Science Center (ASNESC), Northeast Forestry University (Harbin, China) in Songnen Plain in Northeast China. Plants were germinated in water for 3 weeks before stress treatments. Plants were harvested at 6, 12, 24 and 48 h after stress treatments and preserved at −80 °C for real-time PCR.

### 3.2. Phylogenetic Analysis

All protein sequences used for multiple alignments and phylogenetic analysis were extracted from the NCBI non-redundant protein sequences (nr) database using Blastp searches with an e-value cutoff of 1.0 × 10^−6^. The results were used to retrieve proteins with high similarity to the *PutRUB* protein. Then, we used CD search (PMID: 25414356) to analyze the conserved domains within these proteins [47]. Proteins with PDZ/PDZ signaling superfamily or rubredoxin-like superfamily domains were used for further analysis. Alignments of the protein sequences were created with ClustalW (ref) (European Molecular Biology Laboratory, Heidelberg, Germany) using the following parameters: gap opening penalty 10, gap extension penalty 0.2. An unrooted phylogenetic tree was constructed with MEGA 6 (Molecular Evolutionary Genetics Analysis version 6.0, Research Center for Genomics and Bioinformatics, Tokyo Metropolitan University, Hachioji, Tokyo, Japan.) using the maximum likelihood method, and analyses were analysis using the Poisson correction model [48]. We used Gamma distribution to model rate variation sites with shape parameter = 1 distribution to model rate variation sites (shape parameter = 1).

### 3.3. Constructs

The ORF of *PutRUB* was amplified from the *P. tenuiflora* cDNA library using the primers *PutRUB*-*F* and *PutRUB*-*R*. The gene was cloned using *PutRUB*-YE, PB-F and *PutRUB*-YE, PB-R (Table 1). The amplified product was digested with *Bam*HI and *Xho*I, cloned into the plant expression vector pBI121 then sequenced, which was used for subsequent analysis.

To construct GFP fusion proteins, *PutRUB* was amplified with the primers *PutRUB*-GFP-FW and *PutRUB*-GFP-RV (Table 1). The amplified product without the stop codon was digested with *Bam*HI and *Kpn*I, and cloned into the pEGFP vector (Invitrogen, Carlsbad, CA, USA). The constructed plasmid pEGFP-*PutRUB*-GFP was digested with *Bam*HI and *Eco*RI, and cloned into the pYES_2_ vector to obtain the plasmid pYES_2_-*PutRUB*-GFP. Then the product was digested with *Bam*HI and *Sac*I, and cloned into the pBI121 vector to obtain the plasmid pBI121-*PutRUB*-GFP.

The constructs pBI121-*PutRUB*, pBI121-*PutRUB*-GFP and pBI121-GFP (control) were used to generate transgenic Arabidopsis by the floral dip method [49].

### 3.4. Subcellular Localization Assay

pBI121-GFP and pBI121-*PutRUB-*GFP were stably transformed into Arabidopsis. Protoplasts were extracted from transgenic leaves as described in [50]. An Axio Vision fluorescent microscopy system (Axio Imager Z2, Zeiss, Germany) was used to observe fluorescence.

### 3.5. Quantitative Real-Time PCR

Total RNA was extracted by TRIzol reagent (Invitrogen) and cDNA was synthesized with PrimeScript Reverse Transcriptase (Takara Bio, Shiga, Japan) used oligo dT as primer, referring to the manufacturer’s instructions. The cDNA were diluted 5 times with double distilled water for quantitative real-time PCR (qRT-PCR) as templates, with the primers: *PutRUB-QPCR-*F and *PutRUB-QPCR-R*1; Put-Tubulin-F and Put-Tubulin-R. The cDNA was amplified using TransStart Top Green qPCR SuperMix (TransGen Biotech) on a Stratagene Mx3000P QCR system (Agilent Technologies, Cold Spring, NY, USA).The comparative CT method was used to calculated relative expression levels, with the *PutTubulin* gene as an internal control.

### 3.6. Stress Response Analysis in Transgenic Arabidopsis

The plasmid pBI121-*PutRUB* was transformed into Arabidopsis by *A. tumefaciens*-mediated floral dipping. We isolated T_0_-generation seeds of *A. thaliana* on 1/2MS medium containing 50 mg·L^−1^ kanamycin, then collected T_3_ transgenic lines (T3 #1, #2, and #3). Transgenic seedlines were identified by PCR and northern blotting. The CTAB method were used to extract plant genomic DNA [51].

For abiotic stress treatment, WT and T_3_ generation transgenic Arabidopsis seeds were grown on 1/2MS agar plates for 7 days. The seedlings were then transferred to 1/2MS medium supplemented with different chemicals for stress treatments (100 and 125 mM NaCl, 1.5 and 3 mM NaHCO_3_, 2 and 4 mM H_2_O_2_); 1/2MS medium was used as a control. The seedlings were allowed to grow for 10 days (vertical culture, 16/8 h light/dark, temperature 22/18 °C), after which the fresh weights and root growth of the seedlings were measured. Statistical analyses were carried out using Student’s *t*-test.

### 3.7. ROS Detection

Three-week-old transgenic T_2_ generation seedlings of the *PutRUB* were cultivated on 1/2MS medium containing 125 mM NaCl and 3 mM NaHCO_3_ for 12 h at 22 °C, and the *in situ* accumulation of O_2_^−^ was determined by histochemical staining with NBT. Briefly, in an amber-colored bottle, 0.1 g NBT was dissolved in 50 mM sodium phosphate buffer (pH 7.5) and the volume was increased up to 50 mL to get a 0.2% solution. The solution was mixed thoroughly using a magnetic stirrer. The NBT staining solution was prepared fresh before use. The seedlings were placed in test tubes and immersed in NBT staining solution to detect H_2_O_2_. The tubes were wrapped with aluminum foil and keep overnight at room temperature. Then, the chlorophyll was removed for proper visualization of the stain. This was done by immersing the seedlings in absolute ethanol and heating in a boiling water-bath for 10 min (or more if necessary, with intermittent shaking [52].

H_2_O_2_ levels were quantified by the absorbance at a wavelength of 415 nm using titanium sulfate precipitation as described in [53].

## 4. Conclusions

Here, we revealed that a chloroplast-localized rubredoxin family protein from *P. tenuiflora* might be involved in the plant response to environmental stresses. *PutRUB* overexpression increased growth and inhibited H_2_O_2_ accumulation under NaCl and NaHCO_3_ treatments. Our results indicate that *PutRUB* might be involved in maintaining normal electron transfer to enhance transgenic plant adaptability to adversity and reduce ROS accumulation under NaCl and NaHCO_3_ stresses.

## Figures and Tables

**Table 1:** All primers used for PCR analysis.

Primers Name	Sequences (5′→3′)
*PutRUB-*F	ATGGCTGCCACGCTCTCCTCTGT
*PutRUB-*R	CTATTGCAGGCCATATACAAGCAG
*PutRUB*-YE,PB-F	GGATCCATGGCTGCCACGCTCTCCTCTGT
*PutRUB*-YE,PB-R	CTCGAGCTATTGCAGGCCATATACAAGCAG
*PutRUB*-*GFP-*FW	GGTACCATGGCTGCCACGCTCTCCTCTGT
*PutRUB*-*GFP-*RV	GGATCCTTGCAGGCCATATACAAGCAG
*PutRUB-QPCR-*F	GTTCACCAAGACAGCCATCCAG
*PutRUB-QPCR-*R	CAGTCAGCTTCCGTCCAAATCG
Put-Tubulin-F	GTGTCAGCCATACTGTGCCAATC
Put-Tubulin-R	TTGCTCATGCGGTCAGCAATACC
